# Slipknot or Crystallographic Error: A Computational Analysis of the *Plasmodium falciparum* DHFR Structural Folds

**DOI:** 10.3390/ijms23031514

**Published:** 2022-01-28

**Authors:** Rolland B. Tata, Ali F. Alsulami, Olivier Sheik Amamuddy, Tom L. Blundell, Özlem Tastan Bishop

**Affiliations:** 1 Research Unit in Bioinformatics (RUBi), Department of Biochemistry and Microbiology, Rhodes University, Grahamstown 6140, South Africa; ttrolly@gmail.com (R.B.T.); oliserand@gmail.com (O.S.A.); 2 Department of Biochemistry, Sanger Building, University of Cambridge, Tennis Court Rd., Cambridge CB2 1GA, UK; afa32@cam.ac.uk (A.F.A.); tom@cryst.bioc.cam.ac.uk (T.L.B.)

**Keywords:** *P. falciparum* DHFR, PDB, atypical folds, slipknots, crystallographic error

## Abstract

The presence of protein structures with atypical folds in the Protein Data Bank (PDB) is rare and may result from naturally occurring knots or crystallographic errors. Proper characterisation of such folds is imperative to understanding the basis of naturally existing knots and correcting crystallographic errors. If left uncorrected, such errors can frustrate downstream experiments that depend on the structures containing them. An atypical fold has been identified in *P. falciparum* dihydrofolate reductase (*Pf*DHFR) between residues 20–51 (loop 1) and residues 191–205 (loop 2). This enzyme is key to drug discovery efforts in the parasite, necessitating a thorough characterisation of these folds. Using multiple sequence alignments (MSA), a unique insert was identified in loop 1 that exacerbates the appearance of the atypical fold-giving it a slipknot-like topology. However, *Pf*DHFR has not been deposited in the knotted proteins database, and processing its structure failed to identify any knots within its folds. The application of protein homology modelling and molecular dynamics simulations on the DHFR domain of *P. falciparum* and those of two other organisms (*E. coli* and *M. tuberculosis*) that were used as molecular replacement templates in solving the *Pf*DHFR structure revealed plausible unentangled or open conformations of these loops. These results will serve as guides for crystallographic experiments to provide further insights into the atypical folds identified.

## 1. Introduction

Information on the three-dimensional (3D) structure composition of biological macromolecules—particularly proteins and nucleic acids—and their associated ligands and cofactors are archived and managed by the Research Collaboratory for Structural Bioinformatics Protein Data Bank (RCSB PDB) [1,2]. The deposited structures are determined through experimental methods including X-ray crystallography, nuclear magnetic resonance (NMR), or 3D electron microscopy [2].

**Figure 1:**
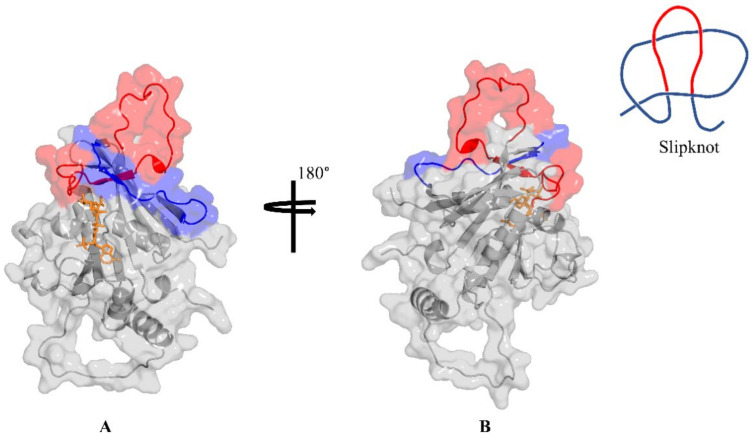
(**A**) The structure of *P. falciparum* DHFR showing loop 1: residues 20–51 (red) and loop 2: residues 191–205 (blue). (**B**) Rotated orientation showing the hind view of (**A**). The topology of a slipknot is shown.

Among the over 160 thousand protein structures currently deposited in the RCSB PDB is a subset of rare proteins with entangled or knotted topologies within their structural folds [3]. Different structural entanglements have been identified, and the protein structures concerned (referred to as knotted proteins) are deposited in the KnotProt database [4,5]. Some of the entangled protein topologies identified include Slipknots (Figure 1), which are knotted subchains that appear unentangled as a whole; probabilistic and deterministic knots including disulfide or ion interactions; cystine knots; and knotoides [5]. The KnotProt database performs regular self-updates, where new PDB entries are scanned for novel entangled topologies. Currently, the database hosts just over 2000 knotted protein entries, more than half of which are slipknots [5]. Although the exact role of protein structural entanglements is unknown, they have been suggested to play important roles in the active site shaping and improvement in overall thermal stability of the protein [3,6].

Besides the knotted proteins in the PDB, another reason for atypical folds in the archived structures is poor structural quality. This has been linked to challenges inherent in the experimental methodology involved in protein structure determination, exacerbated by the complexity of the studied molecules [1,7]. In addition, cognitive bias and flawed epistemology have been identified as the root causes of crystallographic errors [7]. Such errors range from common ones such as wrong atom names and faulty bond angles to major or severe errors such as proteins that are mis-threaded or solved in the wrong space-group [8]. A major reason for severe errors in protein structures is electron density misinterpretation, which may reflect as a violation of stereochemistry, or the occurrence of severe steric clashes in the structure, among others [7].

While the presence of knots and/or severe errors in archived structures in the PDB may be rare, it is important to classify such events once they are identified. For instance, errors in PDB structures may bias the outcome of meta-analyses performed on the structures or cause unusual protein-ligand or antigen-antibody complexing and metal ion binding, among others [7]. On the other hand, proper identification and classification of knotted proteins will enhance further research, leading to insights on the basis of naturally occurring knots in protein structures [3,4,5].

We identified a slipknot-like topology formed between the active site lid-loop (residues 20–51; loop 1) and a second loop (residues 191–205; loop 2) of the *Plasmodium falciparum* DHFR (*Pf*DHFR) enzyme (Figure 1). The *Pf*DHFR enzyme is a well-established drug target and arguably the best for antimalarial drug development [9]. DHFR catalyses the reduction of dihydrofolate to tetrahydrofolate, which is further converted to the one carbon donor methylene tetrahydrofolate, required for the synthesis of the DNA base deoxythymidine monophosphate [10]. Drug discovery efforts targeting this enzyme remain crucial as it is one of only two enzymes that have been targeted successfully from a total of nine established enzymes of the parasites’ folate biosynthesis pathway—giving rise to important antimalarial antifolates pyrimethamine and proguanil [10]. However, these antifolates are both facing resistance, alongside important first line antimalarials such as artemisinin [11,12,13], making it imperative to develop novel antimalarials against established drug targets such as DHFR. The presence of atypical folds in the *Pf*DHFR enzyme, if not well characterised, can therefore frustrate drug discovery efforts targeting the enzyme. This work aims at classifying the identified atypical folds (loop 1 and loop 2) of *Pf*DHFR as either a slipknot or crystallographic error using in silico techniques, including sequence analysis, homology modelling, and molecular dynamics simulations.

## 2. Results and Discussion

### 2.1. Multiple Sequence Alignment Highlights Two Plasmodium Species Specific Inserts in DHFR

**Figure 2:**
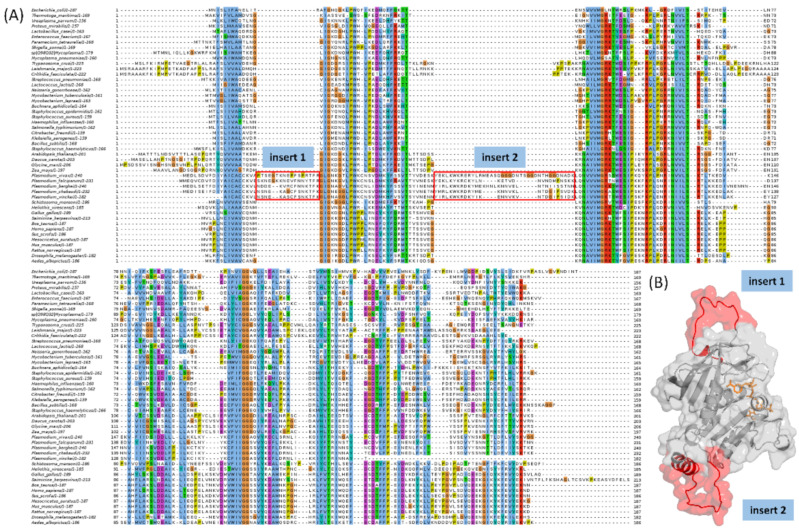
(**A**) Multiple sequence alignment highlighting two *Plasmodium* specific inserts (insert 1 and insert 2) in all of the *Plasmodium* species. (**B**) Structural mapping of the *Plasmodium* specific *P. falciparum* inserts to the modelled 3D structure of the enzyme.

Multiple sequence alignments (MSA) of protein sequences inform the structure, function, and evolutionary relationships across different organisms [14,15]. Prior to MSA, a total of 48 reviewed DHFR and DHFR-TS sequences were downloaded from the Swiss-Prot database [16]. Among these, 12 were from the bifunctional DHFR-TS enzymes. To extract the DHFR subunit from the bifunctional enzymes, MSAs were first performed on the 12 bifunctional enzyme sequences (Figure S1), and using the *Pf*DHFR subunit as a reference, the DHFR sequences of the other enzymes were extracted. Together, all 48 DHFR-only sequences were aligned using PROMALS3D and MUSCLE alignment programs [17,18]. An evaluation of the output MSAs from the two programs in the MUMSA web server [19] revealed that MUSCLE had a slightly higher multiple overlap score (MOS) (0.845012) compared to PROMALS3D (0.841366). This agreed with a visual inspection of both MSAs; hence, the alignment from MUSCLE (Figure 2A) was considered for further interpretation. Overall, the DHFR subunit of *Plasmodium* species shares two unique inserts between residues S22 to R38 (insert 1) and residues Y70 to K97 (insert 2)-numbering adopted from the *Pf*DHFR. While the former consists of a predominantly loop region with a single turn, the latter consists of an alpha helix with three turns (Figure 2B). Both inserts have varying lengths in the *Plasmodium* species. Additionally, insert 1 is located within loop 1 of the identified slipknot-like loops of *Pf*DHFR (Figure 1).

Interestingly, insert 1 of the two human infecting species *P. vivax* and *P. falciparum* is two residues longer than that in the other species *P. berghei*, *P. chabaudi*, and *P. vinckei*. In insert 2, *P. vivax* maintained the longest length, followed by *P. berghei*, while the remaining three shared the same length. To investigate whether the differences in insert lengths could be responsible for host specificity, ten other *Plasmodium* species sequences (including the remaining three human infective species *P. malariae*, *P. ovalae*, and *P. knowlesi*) were downloaded from the Swiss-Prot database and included in the current set for further alignment. However, this alignment was interpreted with caution since the added sequences remain unreviewed. The alignment revealed that insert 1 of the additional three human infecting species shares the same length as the other two human species. However, linking this to host specificity as suggested was not evident since other non-human infecting species shared the same length as those infecting humans, except for *P. yoelii*. The species lengths in insert 2 were more diverse than those in insert 1, which had a conserved length (either 15 or 17 residues each) (Figure S2).

### 2.2. Retrieved DHFR Structures from the PDB Point to a Potentially Misplaced Loop Orientation, Which Is Exacerbated by Insert 1 of Plasmodium Species

We examined all of the *Plasmodium* DHFR structures deposited in the RCSB PDB [1] and noticed a slipknot-like conformation of loop 1 of the enzyme in all structures where this loop was resolved (Table S1). A slipknot, a knotted sub-chain of an entire chain (Figure 1), may be seen topologically as a hairpin-like conformation of a loop region that appears docked onto another loop within the chain [5,6]. Furthermore, when observed in their completed form, slipknots appear untangled but become knotted following the deletion of suitable terminal segments [3]. Knotted proteins are suggested to play roles in the active site shaping and improvement in thermal stability and have been shown to possess loop segments that are absent from the unknotted homologues [3,6]. Since the topology and composition of loops 1 and 2 of *Pf*DHFR fulfil some of the characteristics of slipknots, it was necessary to ascertain if they qualify as such. To achieve this, the KnotProt 2.0 [5] database was searched for the presence of *Pf*DHFR, and the modelled structure of the enzyme (template PDB ID: 4DP3) was also submitted to the KnotProt 2.0 webserver to process for the presence of knots. The results obtained from the database search and the structure processing pointed to the absence of knots or any structural entanglement in the *Pf*DHFR structure. It is worth noting that slipknots generally pose a detection challenge to computational tests that look for knots in complete or full protein structures. However, the algorithm employed by KnotProt 2.0 analyses protein sub-chains [4,6], thus increasing the likelihood to identify slipknots. This suggests that the atypical folds involving loop 1 and loop 2 of *Plasmodium* species DHFR might have resulted from severe crystallographic error: possibly due to mis-threading or wrong space-group solving [8].

**Figure 3:**
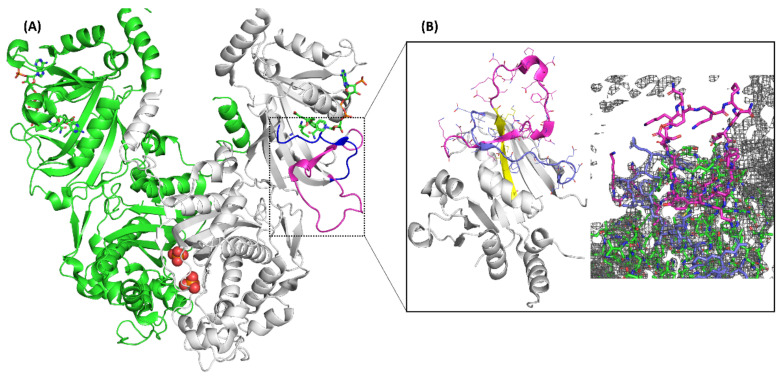
(**A**) Experimental DHFR-TS homodimer structure (PDB ID; 4DP3) coloured in green and white. The NADP cofactor and ligand represented in green stick and phosphate ion as red spheres. Loop 1 is coloured in magenta and loop 2 coloured in blue. A beta-strand clashing with that of loop 1 is coloured in yellow. (**B**) Zoomed in portion showing the absence of electron densities in the experimental structure for loop 1.

The structure determination process by crystallography is known to be liable to error, mainly due to cognitive bias and flawed epistemology [7]. To verify the possibility of loop misplacement in the identified atypical folds of *Pf*DHFR, we began by examining the methods employed in solving one of the earliest crystal structures of *P. falciparum* DHFR-TS homodimer (PDB ID: 4DP3) [11]. While some of the *Pf*DHFR structures in the PDB have missing residues in loop 1 (Table S1), 4DP3 has the completed topology of this loop on both of its DHFR domains (Figure 3A). However, no electron densities were observed for loop 1 in the experimental structure (Figure 3B), which possibly led to incorrect modelling of the strand linked to it.

**Figure 4:**
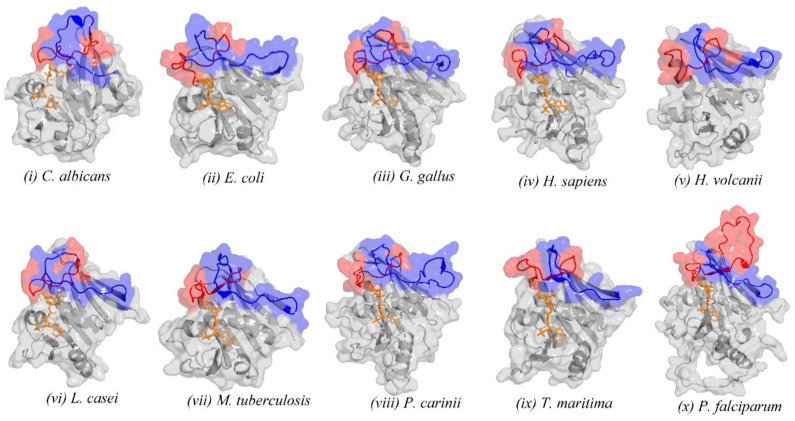
(**i**–**ix**) Structures of the nine molecular replacement templates used in solving the *P. falciparum* DHFR structure, highlighting loop 1 and loop 2. (**x**) Structure of the *P. falciparum* DHFR highlighting loop 1 and loop 2, with the slipknot-like conformation. Loop 1 is coloured in red, and the loop into which it docks (Loop 2) is coloured blue, while the rest of the structure is coloured grey, with the NADP cofactor represented as sticks and coloured in orange.

Interestingly, all nine structures used as molecular replacement templates in solving the DHFR domain of this enzyme possess a similar loop orientation to the 4DP3 structure, albeit with shorter and hence hardly recognisable slipknot-like architecture (Figure 4). A further look at a cross section of DHFR structures deposited in the PDB revealed a similar orientation for all the structures in which this loop was resolved (Table S1).

**Figure 5:**
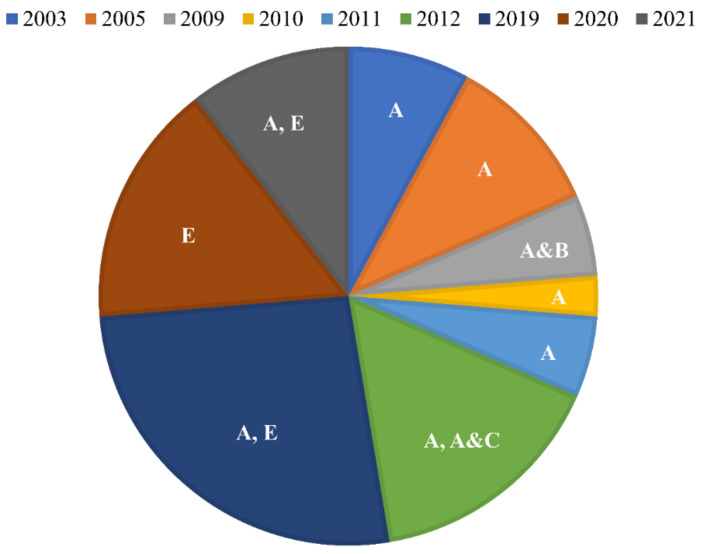
Proportions of *Plasmodium* DHFR structures released by the PDB by year. The different groups responsible for solving the deposited structures are identified by the letters A to E. The use of the comma (,) and ampersand (&) signifies independent and collaborated work among groups in solving the structures released.

The occurrence of errors in crystallographic structures is group or laboratory-specific [7] due partly to the root causes of such errors as highlighted above. We refer to such occurrences here as the lab-effect. To assess possible lab-effects on the deposited *Plasmodium* species DHFR structures in the PDB, we reviewed the author lists of all structures released. Here, the last author’s name was used as an indicator of a specific laboratory or group, leading to the identification of five different groups (A–E) (Figure 5). The structures were classified as coming from the same laboratory if the last authors of the respective author lists were the same. On the other hand, a structure was said to have been solved with the influence from another laboratory if the last author from another author list appeared among the authors of that structure. A search of the PDB identified a total of thirty-eight *Plasmodium* DHFR structures, of which thirty-four were from *P. falciparum* and four were from *P. vivax* (Table S1). The first *Plasmodium* DHFR structures were released in the PDB in 2013 by group A, and this continued over the years till present, despite skipping some years (Figure 5). While group A appears either independently or in collaboration with groups B, C, and D in solving *Plasmodium* DHFR structures over the years, the only group that appears to be not influenced by group A is group E. Interestingly, almost all of the structures released by group A as well as those having their influence possess the complete topologies of loop 1, as opposed to those released by group E, which mainly had missing residues in this loop. Overall, it is likely that group A is at the origin and is mainly responsible for propagating the possible misplacement of loop 1 in the *Plasmodium* DHFR structures deposited in the PDB.

### 2.3. Remodelling the DHFR Structure Using Ab Initio Modelling Programs to Verify Loop Topologies

Differences in loop length and conformation in related protein families are known to be responsible for ligand binding specificity and more, necessitating the accurate modelling of these loops in the different protein structures [20]. To begin with, we submitted the *Pf*DHFR sequence for remodelling in the ab initio modelling program I-TASSER [21] and the recently released artificial intelligence (AI)-based protein structure prediction program AlphaFold [22]. Both programs’ predicted structures were identical to the modelled crystal structures, having RMSDs of 0.292 and 0.468 for I-TASSER and AlphaFold, respectively, with similar loop orientations. However, the observed similarities of these modelled structures to their crystal structures are not unexpected. With I-TASSER, for instance, protein structure databases are first searched for templates, and only the unaligned portions of the query sequence are built from scratch by ab initio folding [21]. Given that several *Plasmodium* DHFR structures deposited in the PDB have completed topologies of both loops 1 and 2, it makes sense for I-TASSER to have retained these topologies for its modelled structure. Second, despite the high accuracy of the AlphaFold program in protein structure predictions, the underlying machine learning (ML) architecture using neural network-based models [22] is dependent on accurate training data sets. AlphaFold uses pair representation from close homologue templates [22], which explains why the loop topologies were retained in its predicted model. Hence, systematic errors from deposited structures in the PDB will most likely affect the accuracy of the predicted structures from downstream structure prediction programs that depend on them for model training as in the case of AI-based programs or as templates for homology-based modelling programs. This will further affect the outcome of molecular docking exercises involving the structures as well as efforts aimed at understanding the mechanism of action of the enzymes and their inhibitors [12,23,24,25].

### 2.4. Evaluating the Conformational Dynamics of the Slipknot-like Loops Using MD Simulations

**Figure 6:**
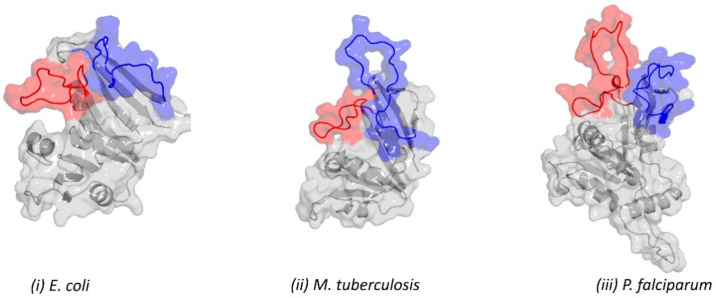
Open conformations of DHFR of: (**i**) *E. coli*, highlighting the separation of the slipknot-like conformation of both loops 1 and 2. (**ii**) *M. tuberculosis*, also showing the separation of both loops 1 and 2. (**iii**) *P. falciparum*, highlighting the separation of the slipknot-like conformation of both loops 1 and 2. All of the structures were obtained from homology modelling, constraining loop 1 to retain the template coordinates while allowing loop 2 to model freely. Colour key: Loop 1 (red), Loop 2 (blue), and the rest of the structure (grey).

In a bid to further explore other possible conformations of loops 1 and 2, we used the homology modelling technique to build two models, each for the *P. falciparum* DHFR, *M. tuberculosis* DHFR, and *E. coli* DHFR structures. The first model was built to retain all of the original crystal structure coordinates for the entire structure (closed conformation), while the second model was built with both loops entirely separated from each other (open conformation). The open conformation was achieved by building an alignment (PIR) file, which constrained the calculated structures to retain the coordinates of loop 1 while allowing loop 2 to be modelled freely (Figure 6). One hundred models were calculated for each structure, and the top model was selected based on the z DOPE and MolProbity scores (Table S2).

The modelled structures were then submitted for triplicate runs of all-atom MD simulations for 200 ns each to assess the conformational evolutions of the structures in both the open and closed conformations of the loops. This was assessed using the protein root mean square deviation (RMSD), the protein root mean square fluctuation (RMSF), the protein radius of gyration (Rg), and the trajectory videos also visualised for interpretation as presented below.

### 2.5. Protein RMSD

**Figure 7:**
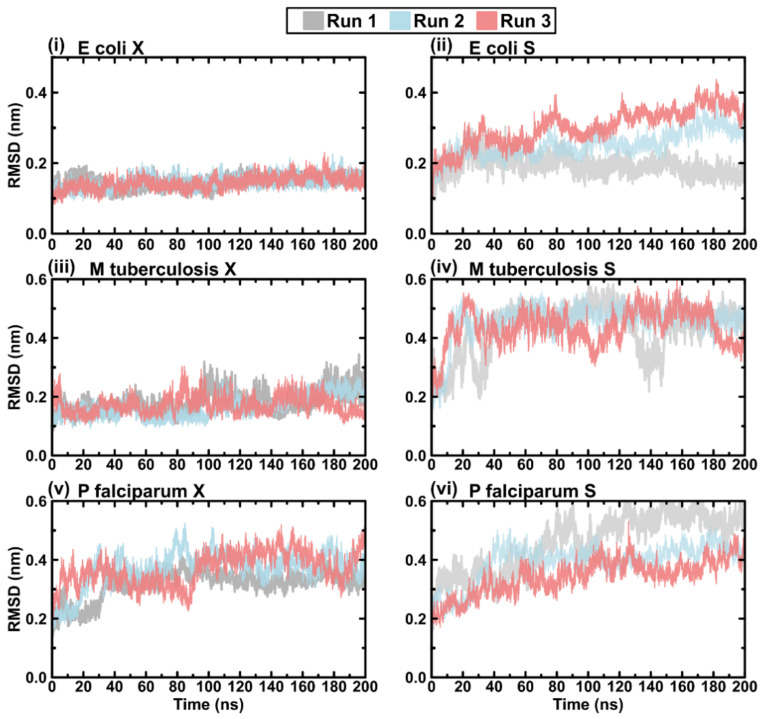
Plots of protein RMSD for the triplicate MD runs: (**i**) the crystal structure models of *E. coli*, (**ii**) the separated loop models of *E. coli*, (**iii**) the crystal structure models of *M. tuberculosis*, (**iv**) the separated loop models of *M. tuberculosis*, (**v**) the crystal structure models of *P. falciparum*, and (**vi**) the separated loop models of *P. falciparum*. Colour key: run 1 (grey), run 2 (blue), and run 3 (red).

Plots of the *E. coli, M. tuberculosis,* and *P. falciparum* RMSDs revealed overall system stabilities for open and closed conformations (Figure 7). The closed conformations plateaued earlier compared with the open conformation, with both maintaining this state throughout the simulation. This is expected since the separated loops in the open conformation require more time for equilibration and energy minimisation compared with the closed conformation. Additionally, the open conformation maintained elevated RMSD values compared with the closed conformation, and this could be due to the relatively higher degree of movement in the separated loops compared with those in the closed conformation.

### 2.6. Protein Rg

**Figure 8:**
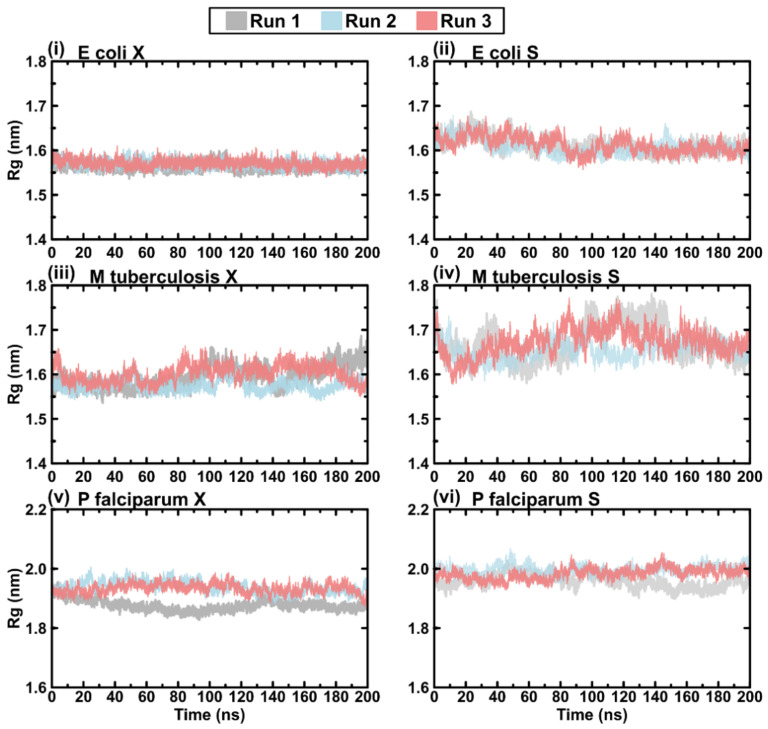
Plots of protein Rg for the triplicate MD runs: (**i**) the crystal structure models of *E. coli*, (**ii**) the separated loop models of *E. coli*, (**iii**) the crystal structure models of *M. tuberculosis*, (**iv**) the separated loop models of *M. tuberculosis*, (**v**) the crystal structure models of *P. falciparum*, and (**vi**) the separated loop models of *P. falciparum*. Colour key: run 1 (grey), run 2 (blue), and run 3 (red).

As seen with RMSD, both the open and closed conformations maintained stable Rg measurements throughout the simulations, and the open conformation was slightly higher than the closed conformation (Figure 8). This represents relatively stable atomic packaging for these conformations throughout the simulation.

### 2.7. Protein RMSF

**Figure 9:**
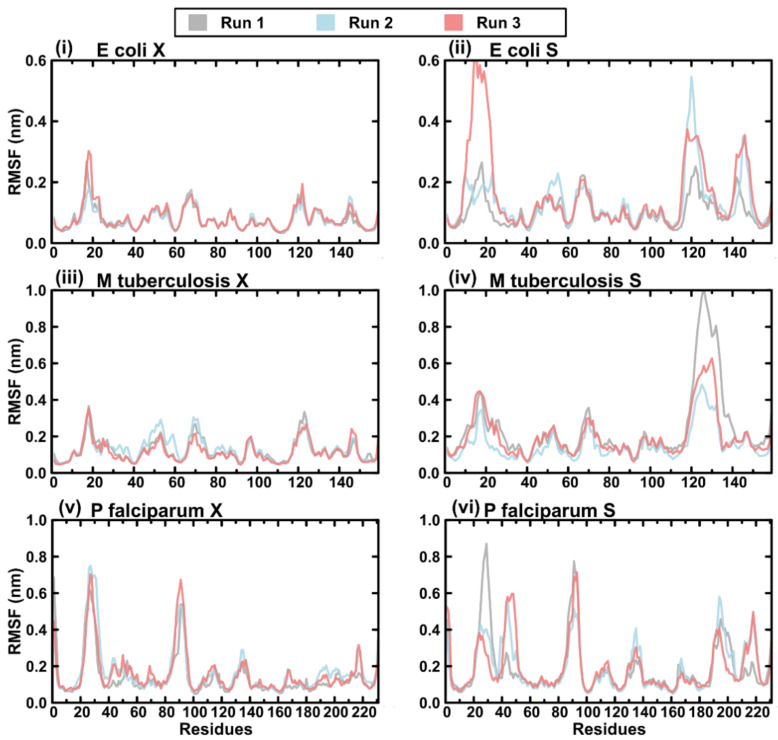
Plots of protein RMSF for the triplicate MD runs: (**i**) the crystal structure models of *E. coli*, (**ii**) the separated loop models of *E. coli*, (**iii**) the crystal structure models of *M. tuberculosis*, (**iv**) the separated loop models of *M. tuberculosis*, (**v**) the crystal structure models of *P. falciparum*, and (**vi**) the separated loop models of *P. falciparum*. Colour key: run 1 (grey), run 2 (blue), and run 3 (red).

Looking at the protein RMSF, the crystal structure models maintained similar fluctuation patterns in all three MD runs, with the highest fluctuations witnessed at the loop regions: for *E. coli*, mainly residues 10–25 (loop 1); for *M. tuberculosis,* mainly residues 9–24 (loop 1) and 116–136 (loop 2); and for *P. falciparum*, mainly residues 20–35 (loop 1) and 85–100 (insert 2) (Figure 9).

For the separated loop conformations, runs 1 and 2 of *E. coli* shared similar degrees of fluctuation in loop 1, while in run three, the loop demonstrated elevated fluctuation levels. This is evident in the trajectory visualisation where this loop almost becomes twisted in run 3, compared with runs 1 and 2, where it remained relatively stable throughout the run (Figure 9i and Videos S1–S6). On the other hand, while similar fluctuation patterns were witnessed with loop two across runs, run one remained more restricted. In *M. tuberculosis*, the only distinction between the closed and open conformations was seen with loop two, which fluctuated the most in run 1 compared with runs 2 and 3 (Figure 9iii,iv). It is worth noting that runs 2 and 3 had the open loops that were transiently closing during the simulation, reminiscent of the crystal structure (closed) conformation. For *P. falciparum*, in addition to fluctuations within loop 1 and insert 2, more fluctuations were noticed involving loop 2 (residues 180–200) alongside two other loops (residues 40–60, and 210–220). Residues 40–60 extended from loop 1 onto an alpha helix that forms part of the active site entrance. Finally, the last loop (residues 210–220) was located on a hairpin extending to the back of the protein structure and is known to form part of the loop subdomain of *E. coli* DHFR [26].

### 2.8. Post MD Structure Evaluation

The comparative PCA method from the MDM-TASK-web web server [27] was used to access representative structures from the different trajectories for further analysis. This method uses the K-means clustering algorithm to arrive at the most accessed conformation within a trajectory after fitting all of the trajectories to a selected reference topology to ensure sampling within a comparable space. Since the trajectories of the triplicate MD runs of the closed conformations had slight deviations from each other (Figure 7), only the first run was retained for comparative PCA. The topologies and trajectories of all three runs of the open conformation were then fitted to this first trajectory of the closed conformation before comparative PCA. An evaluation of the representative structures is shown in Table S2. Overall, it is evident that the quality of all of the structures improved following MD simulations, as portrayed by improvements in the zDOPE and MolProbity scores. Furthermore, the structures with overall quality improvements also portray improved quality scores for loops 1 and 2 (Table S2). Following these evaluations, the representative open conformations were selected from the different runs as follows: *P. falciparum* (run 1), *E. coli* (run 3), and *M. tuberculosis* (run 3).

The pocket volume and druggability scores of the selected structures from MD were evaluated using Fpocket [28]. From this evaluation, it was noticed that the active pocket of *Pf*DHFR became partitioned into two compartments, forming an inner and an outer pocket (Table 1), with a narrow opening linking both compartments. This was seen in both the open and the closed conformations.

This pocket remained open in the whole DHFR-TS dimeric assembly (PDB ID: 4DP3) with volume and druggability scores of 966.363 (0.901) for chain A and 527.641 (0.035) for chain B. The function of loop 1 has been inferred from its positioning in the DHFR-TS dimeric assembly to be responsible for stabilising interdomain interactions between DHFR and TS [11]. Such interactions might maintain a continuous pull on loop 1, hence keeping the active site open as detected in the crystal structure. No partitioning was observed in the active sites of both the open and closed conformations of *E. coli* and *M. tuberculosis*, despite visible increases in the active site volumes of the open conformations. This might be expected since, unlike *Pf*DHFR, both enzymes exist as separate entities from the TS enzymes [11], such that loop 1 does not require anchoring to TS to keep the active site open.

## 3. Materials and Methods

### 3.1. Sequence Retrieval and Multiple Sequence Alignment

Reviewed sequences were obtained from the Swiss-Prot database [16] for the DHFR and the bifunctional DHFR-TS possessing organisms. The Swiss-Prot database comprises manually annotated protein sequences, allocated under the Knowledgebase component of the Universal Protein Resource (UniProt) on protein sequences and functional annotation [16,29]. First, all of the bifunctional DHFR-TS sequences were extracted, and multiple sequence alignment (MSA) was performed. Then, using the DHFR subunit of *P. falciparum* as a reference, the sequences of the DHFR domains of the rest of the bifunctional enzymes were extracted and added to the rest of the DHFR sequences for further alignment. Two sequence alignment programs, MUSCLE [17] and PROMALS3D [18], were utilised, and the best alignment was selected based on visual inspection and the multiple overlap scores (MOS) from the MUMSA web server [19]. While the algorithms used by both MUSCLE and PROMALS3D involve progressive alignment and tree calculations (albeit with distinctive approaches to either), PROMALS3D also incorporates structural information as a guide to the MSA. On the other hand, the MUMSA MOS evaluates the biological correctness of the alignment programs used in MSA. Visualisation of the MSA was achieved in the Jalview MSA editor and analysis workbench [30].

### 3.2. Search for the Presence of Knots

To assess the *Pf*DHFR structure for the presence of any structural entanglements, the protein topology database KnotProt 2.0 [5] was queried for the presence of the structure of *Pf*DHFR. Additionally, the modelled structure of *Pf*DHFR (from PDB ID: 4DP3) was submitted to the structure processing option of the KnotProt 2.0 webserver and assessed for the presence of knots, slipknots, and/or knotoids [5].

### 3.3. Loop Relaxation Using Homology Modelling

For a comprehensive evaluation of the behaviour of the atypical fold investigated in this study, models were built for *E. coli* DHFR (PDB ID: 1RA2), *M. tuberculosis* DHFR (PDB ID: 1DG5), and *P. falciparum* DHFR (PDB ID: 4DP3). The *E. coli* and *M. tuberculosis* DHFRs were chosen from among the nine templates in which the coordinates were used for molecular replacement in the building of the structure of *P. falciparum* during crystallisation. The two loops involved in the atypical fold include residues Val20 to Ile51 and Tyr191 to Tyr205, respectively (Figure 1). Loop 1 forms the hairpin that is docked underneath loop2, leading to the slipknot-like appearance. The homology modelling technique using MODELLER 9.14 software was used to separate both loops before MD simulations. The protein alignment (PIR) file was prepared such that template coordinates of loop 2 were not utilised in the model calculation. This allowed the loop to be modelled freely, thus separating it from loop 1. A total of 100 models were then calculated for each system, and the top-scoring structure was selected based on the zDope and MolProbity scores.

### 3.4. Structure Quality Evaluation

Here, both the template and modelled structural qualities were evaluated by first estimating their normalised Discrete Optimized Protein Energy (zDOPE) scores [31] and then submitting the top-scoring structures for further quality evaluation using the MolProbity 4.4 webserver [32]. MolProbity assesses structure quality using different validation types at the atomic level, including all-atom contact analysis, sidechain rotamers, and Ramachandran backbone criteria.

### 3.5. Molecular Dynamic Simulations

In a bid to attain the optimal topologies of the slipknot-like loops, we performed MD simulations within the Amber forcefield a99SB-disp [33], using the GROMACS v.2018 software package [34]. The a99SB-disp forcefield has been optimised to simulate disordered proteins and maintains high accuracy for folded proteins [33]. This makes it a choice forcefield for use in the simulations envisaged in this study. The GROMACS compatible version of the a99SB-disp forcefield was obtained [35]. All-atom MD simulations were carried out under periodic boundary conditions (PBC). Systems were embedded in explicit TIP4P (a99SBdisp_water) water molecules and enclosed by a cubic simulation box with a clearance space of 1.0 Å from the edges of the protein. Appropriate amounts of Na^+^ and Cl^−^ ions were added to neutralise the total system charges. Before MD simulations, the systems were relaxed by energy minimisation using the steepest descent algorithm with a force threshold of 1000 kJ/mol/nm and a maximum of 50,000 steps. The temperature was equilibrated at 300 K for 100 ps using the modified Berendsen thermostat, according to the canonical-constant number of moles, volume, and temperature (NVT) ensemble. Furthermore, a pressure equilibration was achieved using the Parrinello–Rahman barostat [36], according to the isothermal-isobaric-constant number of moles, pressure, and temperature (NPT) ensemble, to maintain the pressure at 1 bar. During the NVT and NPT equilibration steps, the protein was position restrained using the position restraint algorithm implemented in GROMACS, and constraints were applied to all of the bonds using the LINCS algorithm [37]. Finally, unrestrained production runs were performed in triplicates under periodic boundary conditions for 200 ns each, and the modified Berendsen thermostat as well as the Parinello–Rahman barostat were maintained for temperature and pressure couplings, respectively. In all, the leap-frog integrator was used with an integrator time step of 2 fs, and the Verlet cut-off scheme was implemented using default settings. The coordinates were written at 10.0 ps intervals, and short-range non-bonded contacts (Coulomb and van der Waals interactions) were defined at a 1.4 nm cut-off, while long-range electrostatic interactions were treated using the Particle-mesh Ewald (PME) algorithm [38].

### 3.6. Trajectory Analysis

Prior to completing trajectory analysis, all of the trajectories were corrected for periodic boundary conditions using the *gmx trjconv* tool in GROMACS. This included system centring within the simulation box, fitting the structures to the reference frame to avoid rotational and translational motions and putting back atoms within the box to keep the structures intact. In addition, other GROMACS tools were utilised for calculations as follows: *gmx rms* for root mean square deviation (RMSD), *gmx rmsf* for root mean square fluctuation (RMSF), and *gmx gyrate* for the radius of gyration (Rg). Finally, the k-means clustering algorithm hosted within the comparative PCA option of the MDM-TASK-web webserver [27] was used to obtain representative structures from the different trajectories for structural quality evaluation and analysis.

## 4. Conclusions

Proper characterisation of atypical folds is imperative to the understanding of the basis of naturally occurring knots and correcting crystallisation errors. An atypical fold was identified in the *Pf*DHFR structure, involving residues 20–51 (loop 1) and 191–205 (loop 2). MSA identified a unique insert in loop 1 that exacerbates the appearance of the atypical fold, giving it a slipknot-like topology. However, no knots were detected in the *Pf*DHFR structure and it has not been deposited in the knotted proteins database. Further investigations associated the possible propagation of the identified atypical folds to one research group out of a total of five groups that have deposited *Plasmodium* DHFR structures in the PDB for the past 18 years. The applications of homology modelling and molecular dynamics simulations on the DHFR domain of *P. falciparum* and those of *E. coli* and *M. tuberculosis* that were used as molecular replacement templates in solving its structure revealed plausible unentangled topologies of these loops. These results will guide crystallographic studies on the identified atypical folds of *Plasmodium* DHFR.

## Figures and Tables

**Table 1:** Pocket analysis of the representative structures of both the open and closed conformations of DHFR.

Organism	Conformation	Inner Pocket Volume (Druggability Score)	Outer Pocket Volume (Druggability Score)
*P. falciparum*	OC (run 1)	397.167 (0.003)	617.387 (0.025)
	CC (run 1)	627.592 (0.964)	283.281 (0.006)
*E. coli*	OC (run 3)	1126.757 (0.895)	-
	CC (run 1)	421.624 (0.678)	-
*M. tuberculosis*	OC (run 3)	576.231 (0.851)	-
	CC (run 1)	169.615 (0.001)	-

OC–open conformation, CC–closed conformation.

## Data Availability

All the data is presented in this article and in the Supplementary Materials. Authors are happy to provide the coordinate files of the homology models upon request.

## Supplementary Materials

The following supporting information can be downloaded at: https://www.mdpi.com/article/10.3390/ijms23031514/s1.
