# Supplementary material for: Slipknot or Crystallographic Error: A Computational Analysis of the Plasmodium falciparum DHFR Structural Folds
Source: Int J Mol Sci. 2022 Jan 28;23(3):1514. doi: 10.3390/ijms23031514 (PMC8835989; doi:10.3390/ijms23031514)
Supplement: Supplementary file 1 [file ijms-23-01514-s001.zip › Supplementary_files/Supplementary Table 1.pdf]

Table S1. Reviewed PDB structures.

PDB ID	Release Date	Resolution	Description	Presence of long loop 1	Comment
7CTY	2021-03-31	2.8	Wild type plasmodium falciparum dihydrofolate reductase-thymidylate synthase (PfDHFR-TS), fragment 263, NADP+, dUMP	missing residues	incomplete loop
7CTW	2021-04-28	2.51	Wild-type Plasmodium falciparum dihydrofolate reductase-thymidylate synthase (PfDHFR-TS) complexed with fragment 820, NADPH, dUMP	missing residues	incomplete loop
4DPD	2012-11-14	2.5	WILD TYPE PLASMODIUM FALCIPARUM DIHYDROFOLATE REDUCTASE-THYMIDYLATE SYNTHASE (PfDHFR-TS), DHF COMPLEX, NADP+, dUMP	yes	Complete on chain B not A
3QGT	2011-06-29	2.3	Crystal structure of Wild-type PfDHFR-TS COMPLEXED WITH NADPH, dUMP AND PYRIMETHAMINE	yes	complete on both chains
3UM6	2012-07-18	2.65	Double mutant (A16V+S108T) Plasmodium falciparum DHFR-TS (T9/94) complexed with cycloguanil, NADPH and dUMP	yes	complete on both chains
3UM8	2012-07-18	2.6	Wild-type Plasmodium falciparum DHFR-TS complexed with cycloguanil and NADPH	yes	complete on both chains
3DGA	2009-01-27	2.7	Wild-type Plasmodium falciparum dihydrofolate reductase-thymidylate synthase (PfDHFR-TS) complexed with RJF01302, NADPH, and dUMP	yes	complete having single chains
3DG8	2009-01-27	2.58	Quadruple mutant (N51I+C59R+S108N+I164L) Plasmodium falciparum dihydrofolate reductase-thymidylate synthase (PfDHFR-TS) complexed with RJF670, NADPH, and dUMP	yes	complete having single chains
3QG2	2011-06-29	2.3	Plasmodium falciparum DHFR-TS qradruple mutant (N51I+C59R+S108N+I164L, V1/S) pyrimethamine complex	yes	complete on both chains
1J3K	2003-05-06	2.1	Quadruple mutant (N51I+C59R+S108N+I164L) Plasmodium falciparum dihydrofolate reductase-thymidylate synthase (PfDHFR-TS) complexed with WR99210, NADPH, and dUMP	yes	complete on both chains
1J3J	2003-05-06	2.33	Double mutant (C59R+S108N) Plasmodium falciparum dihydrofolate reductase-thymidylate synthase (PfDHFR-TS) complexed with pyrimethamine, NADPH, and dUMP	yes	complete on both chains
1J3I	2003-05-06	2.33	Wild-type Plasmodium falciparum dihydrofolate reductase-thymidylate synthase (PfDHFR-TS) complexed with WR99210, NADPH, and dUMP	yes	complete on both chains

6A2O	2019-04-24	2.35	Crystal structure of wild type Plasmodium falciparum DHFR-TS complexed with BT3, NADPH, and dUMP	yes	complete on both chains
6A2K	2019-04-24	2.38	Crystal structure of wild type Plasmodium falciparum DHFR-TS complexed with BT1, NADPH, and dUMP	yes	Complete on chain A not B
6A2M	2019-04-24	2.2	Crystal structure of wild type Plasmodium falciparum DHFR-TS complexed with BT2, NADPH, and dUMP	yes	complete on both chains
3UM5	2012-07-18	2.4	Double mutant (A16V+S108T) Plasmodium falciparum dihydrofolate reductase-thymidylate synthase (PfDHFR-TS-T9/94) complexed with pyrimethamine, NADPH, and dUMP	yes	complete on both chains
3JSU	2010-07-28	2.7	Quadruple mutant(N51I+C59R+S108N+I164L) plasmodium falciparum dihydrofolate reductase-thymidylate synthase(PFDHFR-TS) complexed with QN254, NADPH, and dUMP	yes	complete on both chains
6A2P	2019-04-24	2.6	Crystal structure of quadruple mutant (N51I+C59R+S108N+I164L) Plasmodium falciparum DHFR-TS complexed with BT3, NADPH, and dUMP	yes	complete on both chains
6A2L	2019-04-24	2.38	Crystal structure of quadruple mutant (N51I+C59R+S108N+I164L) Plasmodium falciparum DHFR-TS complexed with BT1, NADPH, and dUMP	missing residues	incomplete loop
6A2N	2019-04-24	2.45	Crystal structure of quadruple mutant (N51I+C59R+S108N+I164L) Plasmodium falciparum DHFR-TS complexed with BT2, NADPH, and dUMP	yes	complete on both chains
6KOT	2019-12-04	2.149	Quadruple mutant (N51I+C59R+S108N+I164L) plasmodium falciparum dihydrofolate reductase-thymidylate synthase (PfDHFR-TS) complexed with B12128 and NADPH	missing residues	incomplete loop
6KP7	2019-12-04	1.97	Quadruple mutant (N51I+C59R+S108N+I164L) plasmodium falciparum dihydrofolate reductase-thymidylate synthase (PfDHFR-TS) complexed with B12154	yes	Complete on chain B not A
6KP2	2019-08-14	1.97	Quadruple mutant plasmodium falciparum dihydrofolate reductase complexed with B10042	missing residues	incomplete loop
6KPR	2019-12-04	2.1	Quadruple mutant (N51I+C59R+S108N+I164L) plasmodium falciparum dihydrofolate reductase-thymidylate synthase (PfDHFR-TS) complexed with B12155 inhibitor	missing residues	incomplete loop
6LEZ	2020-12-02	2.644	Quadruple mutant (N51I+C59R+S108N+I164L) plasmodium falciparum dihydrofolate reductase-thymidylate synthase (PfDHFR-TS) complexed with compound 46 and NADPH	missing residues	incomplete loop
6LEV	2020-12-02	2.644	Quadruple mutant (N51I+C59R+S108N+I164L) plasmodium falciparum dihydrofolate reductase-thymidylate synthase (PfDHFR-TS) complexed with compound 46 and NADPH	missing residues	incomplete loop

6LEU	2020-12-02	2.59	Quadruple mutant (N51I+C59R+S108N+I164L) plasmodium falciparum dihydrofolate reductase-thymidylate synthase (PfDHFR-TS) complexed with compound 42 and NADPH	missing residues	incomplete loop
6LHI	2020-12-09	2.59	Quadruple mutant (N51I+C59R+S108N+I164L) plasmodium falciparum dihydrofolate reductase-thymidylate synthase (PfDHFR-TS) complexed with C466 (compound 42) and NADPH	missing residues	incomplete loop
6LH9	2020-12-09	2.644	Quadruple mutant (N51I+C59R+S108N+I164L) plasmodium falciparum dihydrofolate reductase-thymidylate synthase (PfDHFR-TS) complexed with compound 46 and NADPH	missing residues	incomplete loop
6LHJ	2020-11-11	2.4	Quadruple mutant (N51I+C59R+S108N+I164L) plasmodium falciparum dihydrofolate reductase-thymidylate synthase (PfDHFR-TS) complexed with C452 (compound 16) and NADPH	yes	Complete on chain A not B
4DP3	2012-11-14	2.4	Quadruple mutant (N51I+C59R+S108N+I164L) plasmodium falciparum dihydrofolate reductase-thymidylate synthase (PfDHFR-TS) complexed with P218 and NADPH	yes	complete on both chains
4DPH	2012-11-14	2.38	Quadruple mutant (N51I+C59R+S108N+I164L) Plasmodium falciparum dihydrofolate reductase-thymidylate synthase (PfDHFR-TS) complexed with P65 and NADPH	yes	Complete on chain A not B
7F3Y	2021-09-22	2.25	Wild-type Plasmodium falciparum dihydrofolate reductase-thymidylate synthase (PfDHFR-TS) complexed with methotrexate (MTX), NADPH and dUMP	Yes	Complete on chain A and B
7F3Z	2021-09-22	2.6	Double mutant Plasmodium falciparum dihydrofolate reductase-thymidylate synthase (PfDHFR-TS-K1, C59R+S108N) complexed with Trimethoprim (TOP), NADPH and dUMP	Yes	Complete on chain A and B
**Plasmodium vivax (Total of 4 structures)**					
2BL9	2005-09-07	1.9	X-ray crystal structure of Plasmodium vivax dihydrofolate reductase in complex with pyrimethamine and its derivative	yes	Complete and DHFR domain only
2BLC	2005-09-07	2.25	SP21 double mutant P. vivax Dihydrofolate reductase in complex with des-chloropyrimethamine	yes	Complete and DHFR domain only
2BLB	2005-09-07	3	X-ray crystal structure of Plasmodium vivax dihydrofolate reductase in complex with pyrimethamine and its derivative	yes	Complete and DHFR domain only
2BLA	2005-09-07	2.5	SP21 double mutant P. vivax Dihydrofolate reductase in complex with pyrimethamine	yes	Complete and DHFR domain only
**Escherichia coli (Total 58, selected most recent 2010 – 2019, making total of 16 structures)**					
6CQA	2019-01-09	2.2	E. coli DHFR complex with inhibitor AMPQD	no	Complete and DHFR domain only
5UII	2018-01-31	1.351	structure of DHFR with bound buformin and NADP	no	Complete and DHFR domain only

5UIP	2017-11-22	1.9	structure of DHFR with bound DAP, p-ABG and NADP	no	Complete and DHFR domain only
5CCC	2015-08-05	1.5	wild-type E.coli dihydrofolate reductase complexed with 5,10-dideazatetrahydrofolate and oxidized nicotinamide adenine dinucleotide phosphate	no	Complete and DHFR domain only
5CC9	2015-08-05	1.199	L28F E.coli dihydrofolate reductase complexed with 5,10-dideazatetrahydrofolate and oxidized nicotinamide adenine dinucleotide phosphate	no	Complete and DHFR domain only
5UIH	2017-11-22	1.647	structure of DHFR with bound phenformin and NADP	no	Complete and DHFR domain only
5UIO	2017-11-22	1.929	structure of DHFR with bound DAP, p-ABG and NADP	no	Complete and DHFR domain only
5EAJ	2016-09-21	1.701	Crystal structure of DHFR in 0% Isopropanol	no	Complete and DHFR domain only
6MR9	2019-05-15	1.35	E. coli DHFR complex with a reaction intermediate	no	Incomplete and DHFR domain only
4P68	2014-07-16	2.26	Electrostatics of Active Site Microenvironments for E. coli DHFR	no	Complete and DHFR domain only
5UJX	2017-12-27	1.8	Crystal structure of DHFR in 20% Isopropanol	no	Complete and DHFR domain only
