# Supplementary material for: Slipknot or Crystallographic Error: A Computational Analysis of the Plasmodium falciparum DHFR Structural Folds
Source: Int J Mol Sci. 2022 Jan 28;23(3):1514. doi: 10.3390/ijms23031514 (PMC8835989; doi:10.3390/ijms23031514)
Supplement: Supplementary file 1 [file ijms-23-01514-s001.zip › Supplementary_files/Supplementary Table 2.pdf]

Table S2. Structure evaluation for the modelled/template structures and representative structures from MD simulations.

Metric	Region	Close conformation		Open conformation		
		**Original**	**MD1**	**MD1**	**MD2**	**MD3**
**P falciparum**						
MolProbity Score	Whole	2.96	1.57	1.35	1.65	1.57
zDope score	Whole	-0.53	-1.16	-0.746	-0.533	-0.337
Clash>0.4A	Loop1	16	1	0	0	1
	Loop2	9	0	0	0	0
Ramachandran outlier	Loop1	4	6	3	1	7
	Loop2	4	1	3	2	2
Poor Rotamers	Loop1	3	3	2	3	4
	Loop2	3	1	0	1	2
CB deviation outliers	Loop1	0	1	5	3	2
	Loop2	1	0	1	1	1
CaBLAM outliers	Loop1	10	11	9	3	5
	Loop2	8	0	1	5	2
Bond angles outliers	Loop1	5	15	12	14	14
	Loop2	6	7	6	6	5
**E. coli**						
MolProbity Score	Whole	2.38	1.58	1.63	1.67	1.48
zDope score	Whole	-1.14	-1.646	-1.279	-1.366	-1.085
Clash>0.4A	Loop1	10	0	0	1	0
	Loop2	7	2	2	1	0
Ramachandran outlier	Loop1	0	2	2	2	3
	Loop2	0	1	2	3	0
Poor Rotamers	Loop1	1	3	0	1	2
	Loop2	1	1	3	4	2
CB deviation outliers	Loop1	0	0	3	3	2
	Loop2	0	3	1	1	2
CaBLAM outliers	Loop1	4	5	6	4	3
	Loop2	3	3	3	3	3
Bond angles outliers	Loop1	2	6	11	7	4
	Loop2	5	7	9	12	6
**M tuberculosis**						
MolProbity Score	Whole	2.39	1.64	-	2.04	1.59
zDope score	Whole	-1.189	-1.327	-0.842	-0.582	-0.838
Clash>0.4A	Loop1	8	2	-	1	0
	Loop2	6	0	-	0	0
Ramachandran outlier	Loop1	0	2	2	3	3
	Loop2	0	3	2	3	1
Poor Rotamers	Loop1	1	0	5	2	2
	Loop2	2	1	1	4	2
CB deviation outliers	Loop1	0	3	2	0	0
	Loop2	0	3	1	1	2
CaBLAM outliers	Loop1	0	4	3	3	4
	Loop2	2	7	6	5	3
Bond angles outliers	Loop1	0	10	5	6	5
	Loop2	2	9	7	8	12

MD1, MD2, and MD3 represent MD runs 1, 2, and 3 respectively.
