# Supplementary figures and images for: Slipknot or Crystallographic Error: A Computational Analysis of the Plasmodium falciparum DHFR Structural Folds
Source: Int J Mol Sci. 2022 Jan 28;23(3):1514. doi: 10.3390/ijms23031514 (PMC8835989; doi:10.3390/ijms23031514)

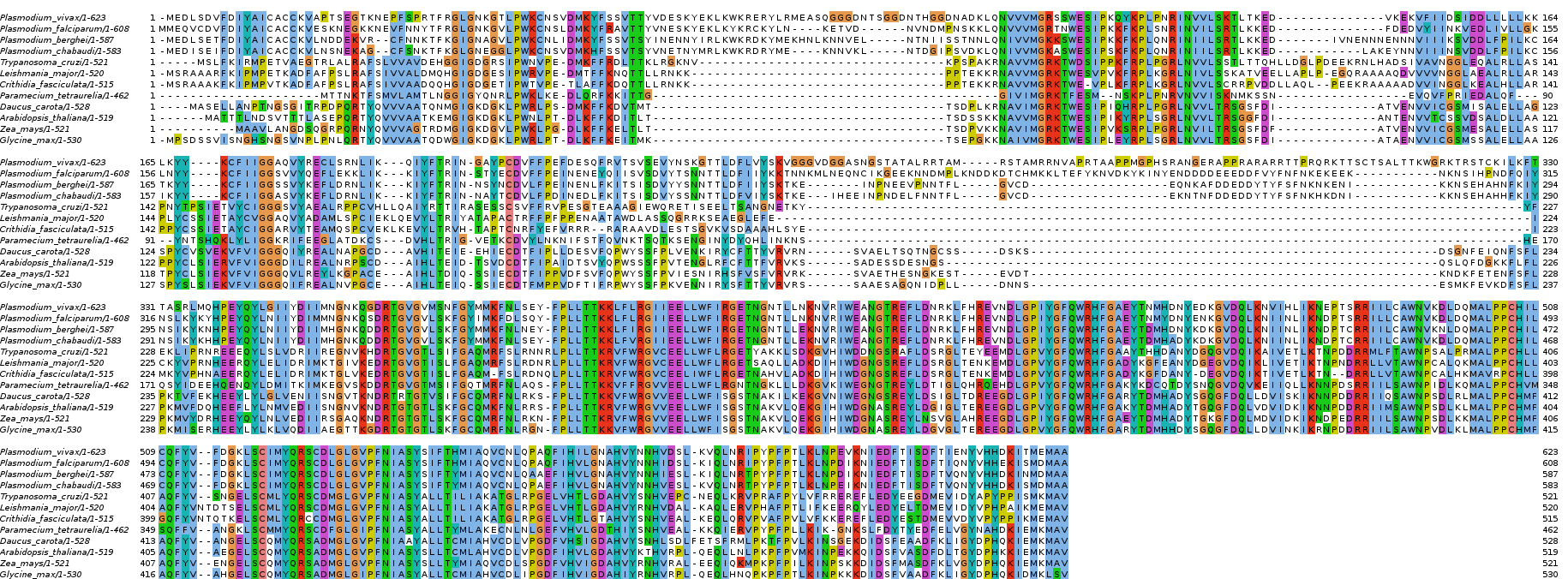

Supplement: Supplementary file 1 [file ijms-23-01514-s001.zip › Supplementary_files/Fig_S1.png]

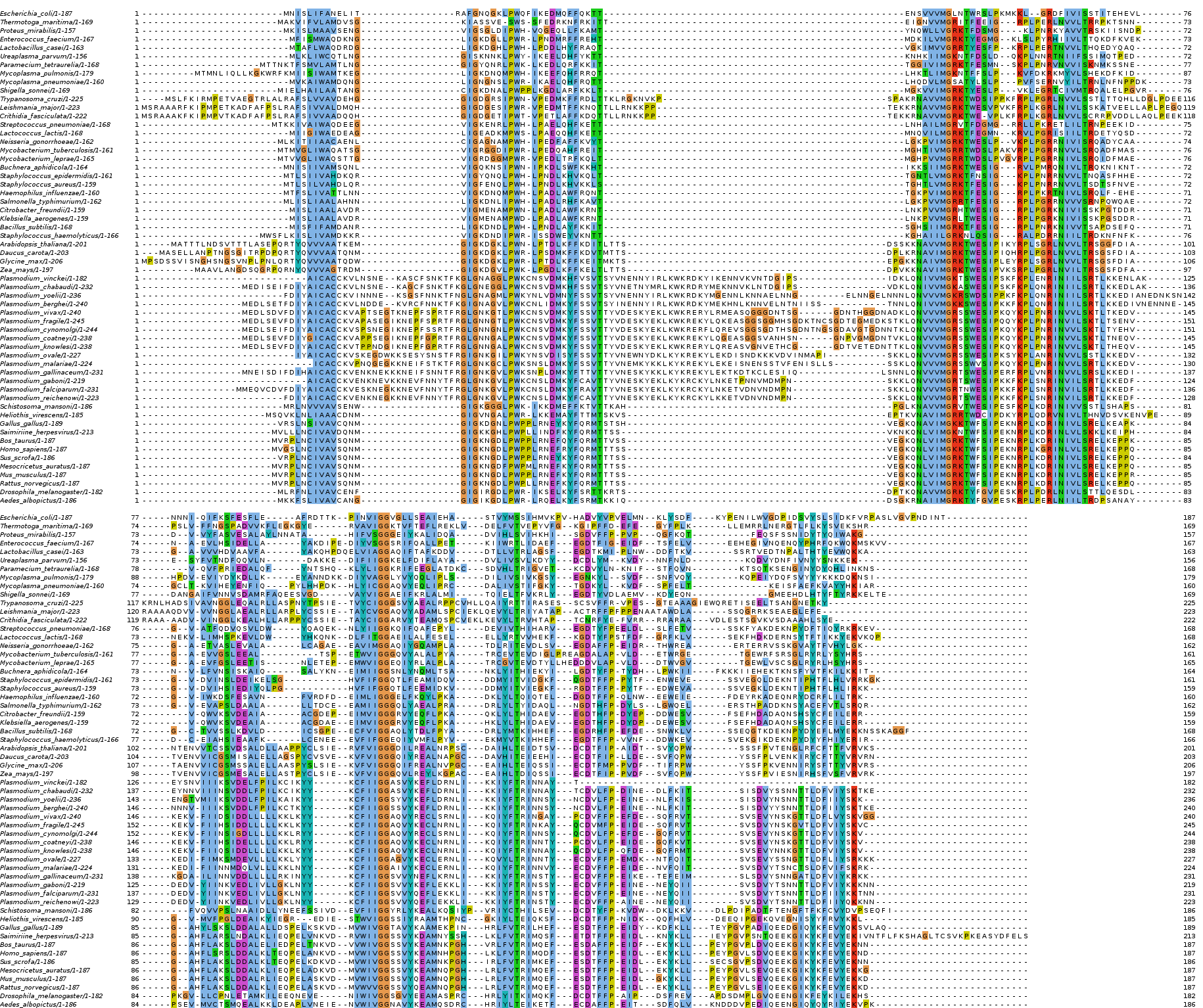

Supplement: Supplementary file 1 [file ijms-23-01514-s001.zip › Supplementary_files/Figure_S2.png]
